# Patient satisfaction after total hip arthroplasty: Influencing factors

**DOI:** 10.3389/fsurg.2022.1043508

**Published:** 2023-01-30

**Authors:** Zhuce Shao, Shuxiong Bi

**Affiliations:** Third Hospital of Shanxi Medical University, Shanxi Bethune Hospital, Shanxi Academy of Medical Sciences, Tongji Shanxi Hospital, Taiyuan, China

**Keywords:** THA – total hip arthroplasty, satisfaction, influence factor, operation, review

## Abstract

It is reported that the dissatisfaction rate after primary total hip arthroplasty (THA) is between 7% and 20%. Patient satisfaction has already become a public health problem that puzzles the world, and it is a problem to be solved that cannot be ignored in the development of global public health. The purpose of this paper is to conduct a narrative review of the literature to answer the following questions: what are the main factors leading to high patient satisfaction or dissatisfaction after THA? The literature on patient satisfaction after THA was reviewed. As far as we know, there is no such detailed and timely overview of THA satisfaction as this article, and the purpose articles we use search engines to search are all RCT (Randomized Controlled Trial) type works, excluding cross-sectional studies and other experiments with low evidence level. Hence, the quality of this article is high. The search engines used are MEDLINE (PubMed) and EMBASE. The keywords used are “THA” and “satisfaction.” The main preoperative, perioperative, and postoperative factors that affect patient satisfaction are summarized in detail below.

## Introduction

Hip osteoarthritis is a common and disabling disease (1–3), and its incidence rate is gradually rising. Total hip arthroplasty (THA) is the most effective procedure to reduce the disability of patients at the end stage (4). This procedure can improve the patient's pain and functional status, even deformity. However, in recent years, although this surgical method has made some significant progress, the study found that many patients still show dissatisfaction after the operation. THA has made crucial technical progress, so future progress in this field may have little impact on patient satisfaction. An emerging area of research is identifying determinants of patient dissatisfaction, which may provide new prospects for improving the quality of care.

## Materials and methods

**Figure 1:**
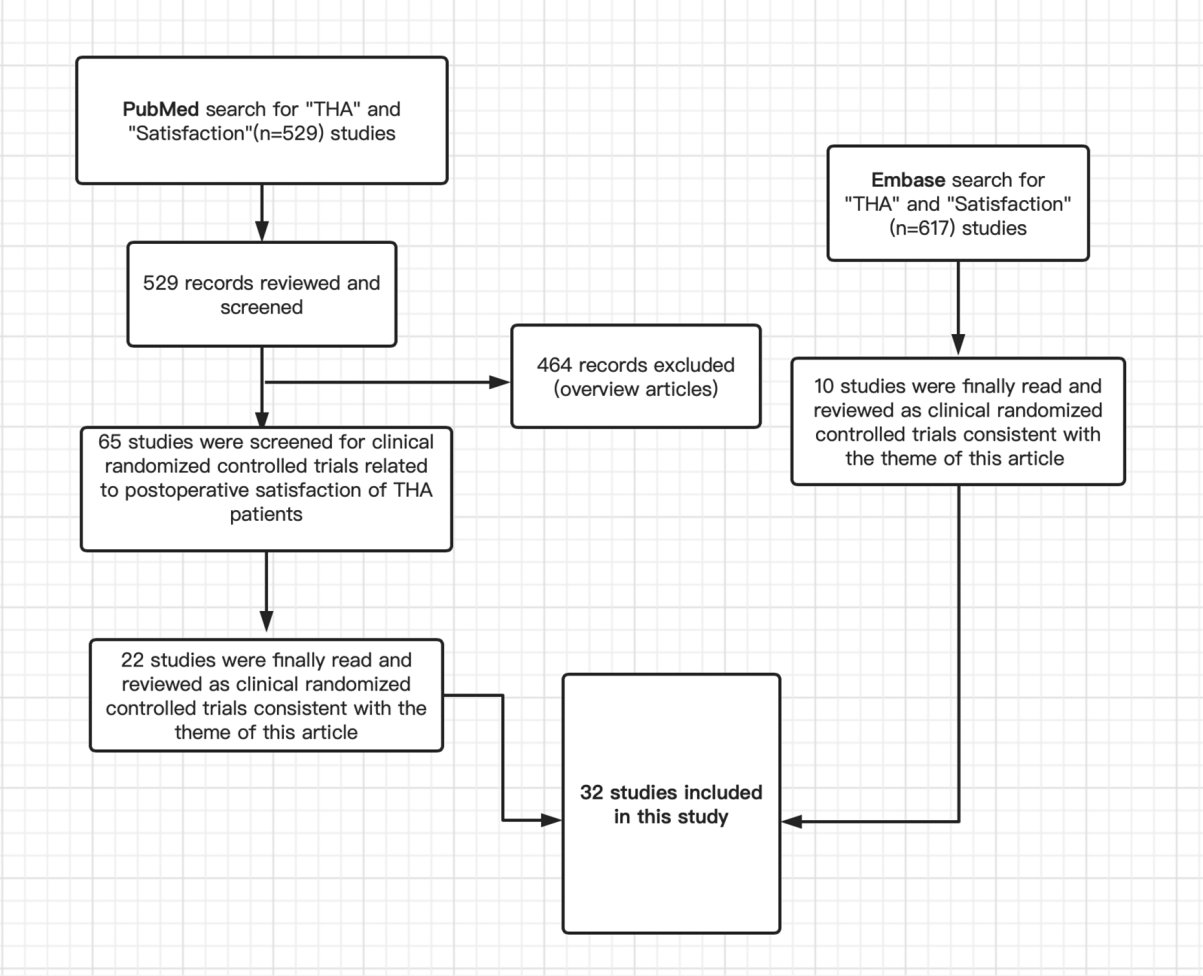
Flowchart.

The literature on patient satisfaction after THA was reviewed. The search engines used are MEDLINE (PubMed) and EMBASE. The keywords used are “THA” and “satisfaction.” The search period includes all available literature on the Internet as of July 13, 2022. Among the 1,146 articles found (529 in PubMed and 617 in EMBASE), 32 articles of RCT (Randomized Controlled Trial) type were selected and reviewed because they were particularly focused on topics (inclusion criteria). In other words, I reviewed those articles on this topic that are particularly important. Figure 1 shows our search strategy.

Some preoperative, perioperative, and postoperative factors may affect patient satisfaction. Classify, analyze and summarize the relevant factors that affect patients’ satisfaction after total hip arthroplasty.

## Results

### Factors influencing postoperative satisfaction of THA patients

Some preoperative, perioperative, and postoperative factors may affect patient satisfaction. We classified them to analyze and summarize the relevant factors that may affect the postoperative satisfaction of THA patients.

### Preoperative factors

Raymond E Anakweet al. conducted a single-center prospective cohort study of 907 consecutive patients with primary THA between 2003 and 2008. The Likert scale was used to evaluate the satisfaction of all patients 12 months after THA. Finally, their study found that preoperative SF-12MCS and a history of depression could predict patient dissatisfaction. Preoperative SF-12MCS and a history of depression were considered predictors of dissatisfaction 1 year after the operation (5).

The research of Mancuso et al. Shows that young patients are most likely to obtain their initial results and may have better satisfaction. Patients with higher preoperative expectations may be more difficult to achieve satisfaction after surgery (6).

Huang Yong et al. Studied type C1 or C2 hip dysplasia and received cementless implant total hip arthroplasty. After at least 10 years of follow-up, C2 had better satisfaction than C1 (88.5–82.8) (7).

Raja Hakim et al. Found that the “minimax” prosthesis, a new generation of short and anatomical femoral shaft manufactured by medacta, successfully reproduced the natural femoral pronation, improved the patient's function and lifestyle, and had better satisfaction than the patients after conventional THA (8).

Chunxue Pu studied the effects of early and late use of celecoxib on the postoperative efficacy, safety, and postoperative satisfaction of patients with hip osteoarthritis undergoing total hip arthroplasty. Their study found that patients in the pre-treatment group who started using celecoxib before THA had better satisfaction than those in the post-treatment group who started using celecoxib after THA (9).

Marina Pinsky et al. Conducted a prospective study. Fifty patients who planned to perform the first THA operation were recruited and divided into intervention groups and control groups. The intervention group received additional structured physical therapy education courses. The results showed that the patients in the intervention group were more satisfied than those in the control group (9.67 ± 0.91 vs. 8.35 ± 1.82, *P* = 0.003) (10).

Hao Li's study is a prospective one. Their primary purpose is to study the effect of duloxetine on postoperative pain and satisfaction with THA. Ninety-six patients were randomly (1,1) assigned to the duloxetine group or placebo group. Finally, it was found that patients using duloxetine had higher postoperative satisfaction (11).

In a particular study by C E h Scott, they adopted the Eurolive dimension (EQ-5D) questionnaire to all patients preparing for replacement surgery, in which EQ-5D < 0 defined the state of “worse than death” (WTD). The study found that patients with WTD before surgery were significantly less satisfied after joint replacement surgery (12).

Süleyman Köro ğ Lu et al. Studied two groups of patients with THA who used a 3-in-1 block of 40 ml of 0.25% bupivacaine and then general anesthesia before the operation only used general anesthesia after simple acupuncture. The final results showed that the patients in the 3-in-1 block 40 ml of 0.25% bupivacaine and the general anesthesia group were more satisfied (13).

The research results of Aggarwal et al. Show that some factors before THA patients can cause dissatisfaction of THA patients, such as lower education and a higher American Association of anesthesiologists (ASA) score will cause lower satisfaction (14).

Duivenvoorden T's prospective study compared the postoperative outcome scores and satisfaction of patients with anxiety or depression with those without anxiety or depression before THA. The final results showed that patients with preoperative anxiety and depression were more likely to be dissatisfied after THA than patients without anxiety or depression (15).

Table 1 summarizes the main preoperative factors that affect patient satisfaction.

**Table 1:** Preoperative factors contributing to patient satisfaction.

Preoperative factors contributing to patient satisfaction positively (+) or negatively (−)			
Authors	Number of patients	Year	Preoperative factors
Raymond et al. (5)	907	2011	Preoperative sf-12mcs and history of depression (−)
Mancuso et al. (6)	180	1997	Young patients (+)
Raja Hakim et al. (8)	19	2022	“Minimax” prosthesis (+)
Chunxue Pu et al. (9)	192	2021	Patients who started using celecoxib before surgery (+)
Marina Pinskiy et al. (10)	50	2021	Received additional structured physical therapy education courses (+)
Hao Li et al. (11)	96	2021	Use duloxetine (+)
C E H Scott et al. (12)	2,073	2019	Preoperative EQ-5D < 0 (−)
Süleyman Köroğlu et al. (13)	30	2008	3 in 1 block 40 ml 0.25% bupivacaine (+)
Aggarwal A et al. (14)	1,412	2022	Lower education and higher American Association of anesthesiologists (ASA) score (−)
Duivenvoorden T et al. (15)	149	2013	Preoperative anxiety and depression symptoms (−)

### Perioperative period

In a prospective comparative randomized study by Ahmed M Samy, the postoperative satisfaction of patients treated with dual mobile THA and standard large head THA was compared. The conclusion shows that THA's dual portable cup design improves patient satisfaction (16).

According to Qiang Xiao's research, the analgesic effect of different layers of the surgical site during primary total hip arthroplasty impacts the postoperative satisfaction of patients. The results show that LIA (local inflammation anesthesia) in deep and superficial fascia and LIA (local inflammation anesthesia) in all layers can significantly improve the postoperative satisfaction of THA patients (17).

In Yiting Lei's randomized blind placebo-controlled trial, we studied whether the fractional dose intravenous dexamethasone regimen was better than the single dose regimen in reducing pain and improving function during the perioperative period of THA. The final results showed that the fractional dose regimen was better than the single dose regimen and could significantly improve patient satisfaction (18).

Qiuru Wang has changed the conventional scar appearance of THA. On the premise of not affecting the recovery of the surgical hand, the bikini incision can improve the subjective satisfaction of patients after THA through DAA (direct anterior approach) (19).

Lingyun Ren's research aims to compare the analgesic effect and satisfaction of preoperative meloxicam and postoperative meloxicam in treating total hip arthroplasty (THA) patients. The research results show that the overall satisfaction of the pre (preoperative analgesia) group is higher than that of the post (postoperative analgesia) group (20).

Chunhua Zhang's research found that the application of rapid surgery combined with the clinical nursing approach in the rehabilitation of patients undergoing total hip arthroplasty can improve the satisfaction of THA patients (21).

Jean Langlois studied the control experiment of using traditional and water fiber absorbable dressings in THA surgery and finally found that patients were more satisfied with the water fiber group (22).

James E Paul et al. Studied whether gabapentin, as an adjuvant for perioperative analgesia of THA, can reduce the use of morphine and improve patient satisfaction. The final results showed that the patient group with gabapentin's adjuvant treatment had worse satisfaction (23).

David Fisher et al. Studied the comparison of absorbable subcutaneous nailing machines and stainless steel wound nailing machines used to close the surgical wound in THA patients during surgery and finally found that patients who used absorbable subcutaneous nailing machines to close the surgical wound had higher satisfaction (24).

Hari K parvataneni's study found that patients with THA who used the multimodal protocol of local periarticular injection had significantly higher satisfaction than the control group (25).

Kong X et al. Studied different auxiliary methods of wound closure in THA and compared the effects of tissue adhesive used for wound closure and standard wound closure methods on the postoperative THA. The final results showed that tissue adhesive could significantly reduce wound drainage and greatly improve patient satisfaction, which can become an ideal auxiliary agent to enhance the recovery of THA standard wound closure (26).

Srampickal G.M. et al. Compared the two different ways of analgesia after THA, which were divided into the periarticular injection of a cocktail of analgesic drugs (PIC) and epidural infiltration (EA) group and epidural infiltration (EA) group. Finally, they found that the overall satisfaction of the PIC group was significantly better than that of the EA group (27).

Table 2 summarizes the main perioperative factors that affect patient satisfaction.

**Table 2:** Perioperative factors contributing to patient satisfaction.

Perioperative factors contributing to patient satisfaction positively (+) or negatively (−)			
Authors	Number of patients	Year	Preoperative factors
Qiang Xiao et al. (17)	120	2021	LIA in deep and superficial fascia and LIA in all layers (+)
Ahmed M Samy et al. (16)	180	2021	Double mobile cup (+)
Yiting Lei et al. (18)	165	2020	Intravenous injection of dexamethasone in different doses (+)
Qiuru Wang et al. (19)	201	2021	Bikini incision (+)
Lingyun Ren et al. (20)	132	2020	Meloxicam was used to relieve pain before operation (+)
Chunhua Zhang et al. (21)	70	2020	Combination of rapid surgery and clinical nursing (+)
Jean Langlois et al. (22)	80	2015	Water fiber absorbable material application (+)
James E Paul et al. (23)	102	2015	Adjuvant therapy of gabapentin (−)
David A Fisher et al. (24)	60	2010	Absorbable subcutaneous nailing machine (+)
Hari K Parvataneni et al. (25)	71	2007	Local periarticular injection multimodal protocol (+)
Kong X et al. (26)	30	2020	Tissue adhesive (+)
Srampickal G.M et al. (27)	50	2019	Periarticular injection of a cocktail of analgesic drugs (PIC) (+)

### Postoperative factors

Jacquelyn Marsh et al. Showed that compared with 90 patients (75.6%) in the online follow-up group, 91 patients (82.0%) in the general care group were very satisfied with the follow-up process (28).

Maria Grazia Benedetti designed a prospective study to divide patients after THA into a study group and a control group. The study group will receive an additional 2 weeks of rehabilitation to strengthen the abductor's muscles. Finally, it was found that in addition to standard repair, supporting the rehabilitation plan of hip muscles of patients receiving THA determined increased muscle strength, improving functional performance and patient satisfaction (29).

Hirose Shakya's prospective study found that after THA patients began to take zolpidem after surgery, their quality of life and satisfaction would be significantly improved (30).

Kimona Issa's study finally showed that young patients were more likely to be dissatisfied with physiotherapy after THA (31).

Kukreja P et al. Studied the comparison of analgesic methods after THA. They compared the Quadratus lumborum (QL) block group and the non-QL block group. Finally, they found that patients who used Quadratus lumborum (QL) block after THA had significantly higher satisfaction (32).

Melson T et al. Did a prospective study. Among them, the sufentanil sublingual tablet computer system (ZalvisoTM) is a handheld PCA device that can provide sufentanil 15 microgram tablets (SST15). The locking time is 20 min, allowing patients to start at their comfort level. In addition, the standard intravenous patient control analgesia (IV PCA) was used as a control experiment. Finally, it was found that compared with IV PCA MS, Patients with sst15 are more satisfied (33).

Wang's prospective controlled trial studied the effect of the automated intermittent boluses group with continuous ultrasound-guided fascia iliaca compartment block (FICB) after total hip arthroplasty and compared it with the constant infusion group. The results showed that the satisfaction of the automated intermittent boluses group was higher after analgesia (34).

The purpose of the study of Ganathy K.M. et al. Is to evaluate that after THA, unrestricted functional activities can be allowed, and patients can resume exercise and daily activities without restrictions, including squatting and cross-legged sitting. Patients who need complete joint replacement have higher expectations than in the past and often go far beyond improving pain relief and mobility. The final results showed that the satisfaction of unrestricted patients was higher (35).

Harper C.M. et al. Evaluated the role of animal adjuvant therapy with therapy dogs in the postoperative rehabilitation of patients with THA. Through a randomized controlled study, the final results showed that the use of treatment dogs positively affected the satisfaction of patients after total hip arthroplasty (36).

In a prospective controlled trial conducted by Singelyn F.J. and others, to evaluate the impact of the most appropriate postoperative analgesia technology after THA on the postoperative performance of THA, the study population was divided into three groups: intravenous (IV) patient-controlled analgesia (PCA) and morphine group, continuous “3 in 1” block group, and patient-controlled epidural analgesia (PCEA) group. The final results showed that patients in the constant “3 in 1” block group had the highest satisfaction (37).

Table 3 summarizes the main postoperative factors that affect patient satisfaction.

**Table 3:** Postoperative factors contributing to patient satisfaction.

Postoperative factors contributing to patient satisfaction positively (+) or negatively (−)			
Authors	Number of patients	Year	Preoperative factors
Jacquelyn marsh et al. (28)	229	2014	General nursing after operation (+)
Maria Grazia Benedetti et al. (29)	103	2021	Postoperative hip muscle rehabilitation plan (+)
Hirose Shakya et al. (30)	160	2019	After taking zolpidem after operation (+)
Kimona Issa et al. (31)	100	2013	Physical therapy for young patients after surgery (−)
Kukreja P et al. (32)	80	2019	Quadratus lumborum (QL) block (+)
Melson T et al. (33)	84	2016	Provide sufentanil 15 microgram tablets (SST15) (+)
Wang N et al. (34)	60	2016	The automated intermittent boluses group with continuous ultrasound-guided fascia iliaca compartment block (FICB) (+)
Ganapathy K.M et al. (35)	196	2016	Unrestricted functional activities after operation (+)
Harper C.M et al. (36)	72	2015	Use treatment dogs (+)
Singelyn F.J et al. (37)	1,338	1999	Continuous “3 in 1” block (+)

## Discussion

THA is one of the most frequent operations in the world. Most patients benefit from primary THA by reducing hip pain, improving function, and improving quality of life. Despite these fact-based improvements, it is reported that satisfaction after TKA is very high; However, other studies have shown that patients’ satisfaction after primary THA is not well guaranteed.

In recent years, clinicians and patients have paid more attention to patients’ satisfaction after surgery, and postoperative satisfaction has gradually become a criterion for evaluating surgery. The influencing factors of postoperative satisfaction of patients with THA are always diverse, which may not be the influence of a single element, but the combination of multiple factors. Therefore, we should pay as much attention to some factors that affect patients’ satisfaction with THA before, during, and after surgery, and may intervene to make the satisfaction develop in a good direction.

We have noticed that many factors that affect the postoperative satisfaction of THA patients are related to their state, such as psychological factors, whether they are depressed or anxious, whether they are young, etc., so it is also advisable to conduct a simple patient evaluation before surgery and make correct intervention according to the patient groups that are prone to dissatisfaction.

In addition, many factors that affect the postoperative satisfaction of THA patients are related to the existing methods or technologies of THA surgery, so continue the sustainable development of scientific research achievements and strive to develop better surgical methods to facilitate patients to obtain better treatment and higher satisfaction.

Finally, we also found that many factors that affect the postoperative satisfaction of THA patients have a significant relationship with postoperative pain, so we must pay attention to the individualized analgesic treatment promptly according to the different conditions of patients after the operation to achieve higher satisfaction and comfort of patients so that patients can spend a period of acute pain after the operation smoothly and without special pain.

Some preoperative, perioperative, and postoperative factors contribute to patient satisfaction. Identifying patients at risk of dissatisfaction is valuable for counseling and education and may reduce the overall rate of dissatisfied patients. Nevertheless, further research is needed to develop a simple but reliable questionnaire to predict patients’ satisfaction after primary THA consistently.

## Conclusion

Patient satisfaction after THA is associated with various factors affecting the patient preoperatively, postoperatively, and perioperatively. Surgeons need to reduce the gap between surgeon and patient expectations and improve patient satisfaction after THA so that they can better accept the procedure's outcome and facilitate good postoperative psychology and further recovery. This requires consideration of a wide range of factors related to their outcomes. Our study and this article attempt to better review and understand the influences related to patient satisfaction after THA. In the future, studies using novel tools to assess these factors will contribute to a better understanding of patient satisfaction after THA.

## Data Availability

The original contributions presented in the study are included in the article/Supplementary Material, further inquiries can be directed to the corresponding author.

## References

[1] Picavet HS Hazes JM. Prevalence of self reported musculoskeletal diseases is high. Ann Rheum Dis. (2003) 62(7):644–50. 10.1136/ard.62.7.644 12810427 PMC1754612

[2] Woolf AD Pfleger B. Burden of major musculoskeletal conditions. Bull World Health Organ. (2003) 81(9):646–56. PMID: 14710506; PMCID: PMC2572542 14710506 PMC2572542

[3] Pereira D Peleteiro B Araújo J Branco J Santos RA Ramos E. The effect of osteoarthritis definition on prevalence and incidence estimates: a systematic review. Osteoarthr Cartil. (2011) 19(11):1270–85. 10.1016/j.joca.2011.08.009 21907813

[4] Higashi H Barendregt JJ. Cost-effectiveness of total hip and knee replacements for the Australian population with osteoarthritis: discrete-event simulation model. PLoS One. (2011) 6(9):e25403. 10.1371/journal.pone.0025403 21966520 PMC3179521

[5] Anakwe RE Jenkins PJ Moran M. Predicting dissatisfaction after total hip arthroplasty: a study of 850 patients. J Arthroplasty. (2011) 26(2):209–13. 10.1016/j.arth.2010.03.013 20462736

[6] Mancuso CA Salvati EA Johanson NA Peterson MG Charlson ME. Patients’ expectations and satisfaction with total hip arthroplasty. J Arthroplasty. (1997) 12(4):387–96. 10.1016/s0883-5403(97)90194-7 9195314

[7] Huang Y Zhou Y Shao H Chu Y Gu J Li H. Total hip arthroplasties for hartofilakidis type C1 and C2 high hip dislocations demonstrate similar survivorship and clinical function at minimum 10-year follow-up with cementless implants. J Arthroplasty. (2022) 37(12):2374–80. 10.1016/j.arth.2022.06.005 35709909

[8] Hakim R Weinstein A Dabby D Rozen N Shabshin N Rubin G. Successful reconstruction of natural femoral anteversion using a short femoral stem in total hip arthroplasty surgery. J Int Med Res. (2022) 50(4):3000605221091500. 10.1177/03000605221091500 35443831 PMC9047853

[9] Pu C Jiang X Sun Y Lin H Li S. Efficacy and safety between early use and late use of celecoxib in hip osteoarthritis patients who receive total hip arthroplasty: a randomized, controlled study. Inflammopharmacology. (2021) 29(6):1761–8. 10.1007/s10787-021-00880-1 34727277

[10] Pinskiy M Lubovsky O Kalichman L. The effect of a preoperative physical therapy education program on short-term outcomes of patients undergoing elective total hip arthroplasty: a controlled prospective clinical trial. Acta Orthop Traumatol Turc. (2021) 55(4):306–10. 10.5152/j.aott.2021.20108 34464304 PMC12462783

[11] Li H Zeng WN Ding ZC Yuan MC Cai YR Zhou ZK. Duloxetine reduces pain after total hip arthroplasty: a prospective, randomized controlled study. BMC Musculoskelet Disord. (2021) 22(1):492. 10.1186/s12891-021-04377-4 34049519 PMC8161627

[12] Scott CEH MacDonald DJ Howie CR. ‘Worse than death’ and waiting for a joint arthroplasty. Bone Joint J. (2019) 101-b(8):941–50. 10.1302/0301-620x.101b8.Bjj-2019-0116.R1 31362549 PMC6681678

[13] Köroğlu S Takmaz SA Kaymak C Narli A Karalezli K Dikmen B. The preoperative analgesic effect of 3-in-1 block on postoperative pain and tramadol consumption in total hip arthroplasty. Agri. (2008) 20(1):19–25. PMID: 18338275 18338275

[14] Aggarwal A Naylor JM Adie S Liu VK Harris IA. Preoperative factors and patient-reported outcomes after total hip arthroplasty: multivariable prediction modeling. J Arthroplasty. (2022) 37(4):714–720.e4. 10.1016/j.arth.2021.12.036 34990754

[15] Duivenvoorden T Vissers MM Verhaar JAN Busschbach JJV Gosens T Bloem RM Anxiety and depressive symptoms before and after total hip and knee arthroplasty: a prospective multicentre study. Osteoarthr Cartil. (2013) 21(12):1834–40. 10.1016/j.joca.2013.08.022 24012622

[16] Samy AM Mahmoud AA El-Tantawy A. Dual mobility cup: does it improve patient's satisfaction after total hip arthroplasty? A prospective comparative randomized study. J Am Acad Orthop Surg. (2021) 29(22):e1141–50. 10.5435/jaaos-d-20-00882 33252552

[17] Xiao Q Xu B Wang H Luo Z Yuan M Zhou Z Analgesic effect of single-shot ropivacaine at different layers of the surgical site in primary total hip arthroplasty: a randomised, controlled, observer-blinded study. J Orthop Surg Res. (2021) 16(1):81. 10.1186/s13018-020-02182-8 33482850 PMC7821717

[18] Lei Y Huang Z Huang Q Pei F Huang W. Is a split-dose intravenous dexamethasone regimen superior to a single high dose in reducing pain and improving function after total hip arthroplasty? A randomized blinded placebo-controlled trial. Bone Joint J. (2020) 102-b(11):1497–504. 10.1302/0301-620x.102b11.Bjj-2020-1078.R1 33135436

[19] Wang Q Yue Y Yang Z Chen L Li Q Kang P. Comparison of postoperative outcomes between traditional longitudinal incision and bikini incision in total hip arthroplasty via direct anterior approach: a randomized controlled trial. J Arthroplasty. (2021) 36(1):222–30. 10.1016/j.arth.2020.07.047 32800438

[20] Ren L Meng L Yan H Sun W Yao D. Preoperative meloxicam versus postoperative meloxicam for pain control, patients’ satisfaction and function recovery in hip osteoarthritis patients who receive total hip arthroplasty: a randomized, controlled study. Inflammopharmacology. (2020) 28(4):831–8. 10.1007/s10787-020-00718-2 32506275 PMC7363719

[21] Zhang C Xiao J. Application of fast-track surgery combined with a clinical nursing pathway in the rehabilitation of patients undergoing total hip arthroplasty. J Int Med Res. (2020) 48(1):300060519889718. 10.1177/0300060519889718 31939326 PMC7254164

[22] Langlois J Zaoui A Ozil C Courpied JP Anract P Hamadouche M. Randomized controlled trial of conventional versus modern surgical dressings following primary total hip and knee replacement. Int Orthop. (2015) 39(7):1315–9. 10.1007/s00264-015-2726-6 25787680

[23] Paul JE Nantha-Aree M Buckley N Shahzad U Cheng J Thabane L Randomized controlled trial of gabapentin as an adjunct to perioperative analgesia in total hip arthroplasty patients. Can J Anaesth. (2015) 62(5):476–84. 10.1007/s12630-014-0310-y 25772701

[24] Fisher DA Bengero LL Clapp BC Burgess M. A randomized, prospective study of total hip wound closure with resorbable subcuticular staples. Orthopedics. (2010) 33(9):665. 10.3928/01477447-20100722-12 20839703

[25] Parvataneni HK Shah VP Howard H Cole N Ranawat AS Ranawat CS. Controlling pain after total hip and knee arthroplasty using a multimodal protocol with local periarticular injections: a prospective randomized study. J Arthroplasty. (2007) 22(6 Suppl 2):33–8. 10.1016/j.arth.2007.03.034 17823012

[26] Kong X Yang M Cao Z Chen J Chai W Wang Y. Tissue adhesive for wound closure in enhanced-recovery total hip arthroplasty: a prospective, randomized and controlled study. BMC Musculoskelet Disord. (2020) 21(1):178. 10.1186/s12891-020-03205-5 32192465 PMC7083038

[27] Srampickal GM Jacob KM Kandoth JJ Yadev BK Palraj T Oommen AT How effective is periarticular drug infiltration in providing pain relief and early functional outcome following total hip arthroplasty? J Clin Orthop Trauma. (2019) 10(3):550–4. 10.1016/j.jcot.2018.06.005 31061588 PMC6492212

[28] Marsh J Bryant D MacDonald SJ Naudie D Remtulla A McCalden R Are patients satisfied with a web-based followup after total joint arthroplasty? Clin Orthop Relat Res. (2014) 472(6):1972–81. 10.1007/s11999-014-3514-0 24562873 PMC4016458

[29] Benedetti MG Cavazzuti L Amabile M Tassinari E Valente G Zanotti G Abductor muscle strengthening in THA patients operated with minimally-invasive anterolateral approach for developmental hip dysplasia. Hip Int. (2021) 31(1):66–74. 10.1177/1120700019877174 31544524

[30] Shakya H Wang D Zhou K Luo ZY Dahal S Zhou ZK. Prospective randomized controlled study on improving sleep quality and impact of zolpidem after total hip arthroplasty. J Orthop Surg Res. (2019) 14(1):289. 10.1186/s13018-019-1327-2 31481074 PMC6724364

[31] Issa K Naziri Q Johnson AJ Memon T Dattilo J Harwin SF Evaluation of patient satisfaction with physical therapy following primary THA. Orthopedics. (2013) 36(5):e538–42. 10.3928/01477447-20130426-12 23672902

[32] Kukreja P MacBeth L Sturdivant A Morgan CJ Ghanem E Kalagara H Anterior quadratus lumborum block analgesia for total hip arthroplasty: a randomized, controlled study. Reg Anesth Pain Med. (2019) 44(12):1075–9. 10.1136/rapm-2019-100804 31653800

[33] Melson T Turan A Minkowitz H Di Donato K Palmer P. Sufentanil sublingual tablet 15mcg vs IV PCA morphine: a comparative analysis of patient satisfaction and drug utilization by surgery type. Reg Anesth Pain Med. (2016) 41(5):e85. 10.1097/AAP.0000000000000469

[34] Wang N Li M Geng J Chen X Guo X. A clinical study of the efficacy of automated intermittent boluses for continuous fascia iliaca block. Natl Med J China. (2016) 96(22):1750–4. 10.3760/cma.j.issn.0376-2491.2016.22.008 27356642

[35] Ganapathy KM Nandkumar Sundaram S. Activity restrictions after tja: are they essential? Osteoporos Int. (2016) 27(SUPPL 1):S215. 10.1007/s00198-016-3530-x

[36] Harper CM Dong Y Thornhill TS Wright J Ready J Brick GW Can therapy dogs improve pain and satisfaction after total joint arthroplasty? A randomized controlled trial. Clin Orthop Relat Res. (2015) 473(1):372–9. 10.1007/s11999-014-3931-0 25201095 PMC4390934

[37] Singelyn FJ Gouverneur JMA. Postoperative analgesia after total hip arthroplasty: IV PCA with morphine, patient-controlled epidural analgesia, or continuous ‘3-in-1’ block?: a prospective evaluation by our acute pain service in more than 1,300 patients. J Clin Anesth. (1999) 11(7):550–4. 10.1016/S0952-8180(99)00092-6 10624638

